# *Pyrus calleryana* extracts reduce germination of native grassland species, suggesting the potential for allelopathic effects during ecological invasion

**DOI:** 10.7717/peerj.15189

**Published:** 2023-04-25

**Authors:** Michaela J. Woods, Jonathan T. Bauer, Dena Schaeffer, Ryan W. McEwan

**Affiliations:** 1 Biology Department, University of Dayton, Dayton, OH, United States of America; 2 Department of Biology and the Institute for the Environment and Sustainability, Miami University of Ohio, Oxford, OH, United States of America

**Keywords:** Bradford pear, Callery pear, Phenology, Prairie, Biological invasion

## Abstract

Invasive plant species’ success may be a result of allelopathy, or the release of secondary metabolites that are harmful for surrounding plant species. Allelopathy can be mediated through the abiotic environment by chemical sorption or transformation, so the substrate on which interactions occur can lead to differential outcomes in allelopathic potential. One aggressive invader, *Pyrus calleryana*, has become dominant in many ecosystems throughout Eastern US, and has reduced the abundance of native species where it invades. Thus, our goal was to identify if *P. calleryana* had allelopathic potential by testing the impact of leaf and flower leachate on gemination of six common grassland species (three grasses and three forbs) in either sterilized sand or field collected soils. Germination of five out of six tested species was reduced by *P*. *calleryana* leaf litter, with weaker impacts from flower leachate. This suggests that allelopathy is one mechanism driving the success of *P. calleryana* and that allelopathic effects may change with plant phenology. For instance, *P. calleryana* has late leaf senescence in the fall and copious blooming in the spring that may elongate the timeframe that allelopathic inhibition can occur. Further, germination was higher in sand than in soil, suggesting that the context of the abiotic environment can mediate this relationship. In our study, two grass species that could be overabundant in restored grasslands had higher germination rates in soil than sand and one was not altered by *P. calleryana* suggesting that this relationship could further promote the overabundance of grass species. Taken together, *P. calleryana* likely inhibits the germination of native species where it invades, but there is context dependency of this relationship with both soil chemistry and seasonality.

## Introduction

Non-native invasive plants can displace native plant species, reducing biodiversity (Gioria & Osborne, 2014). One mechanism conferring an advantage for some invasive species is allelopathy, the release of biochemicals that negatively affect other plants directly or indirectly through alterations in the soil microbial community (Callaway & Ridenour, 2004; Inderjit et al., 2011). The majority of tested invasive plant species are allelopathic (Kalisz, Kivlin & Bialic-Murphy, 2021), suggesting that the role of allelopathy in promoting species invasion is likely widespread. Allelopathy can influence plants at different life stages and may be identified by a reduction in germination rates or plant growth (Zhang et al., 2021). For example, the invasive shrub *Lonicera maackii* reduced seed germination rates of some native grassland and forest plant species (McEwan et al., 2010; Bauer et al., 2012), which has the potential to influence longer-term community assembly. This may be particularly important in early successional ecosystems where the trajectory of plant communities can be substantially altered due to allelopathic invasive plants that prevent native plant establishment (Cramer, Hobbs & Standish, 2008). Thus, identifying the interactions between invasive plants and the germination of native seeds can elucidate the impacts of invasion on native plant communities in ecosystems that are prone to invasion.

The relative impacts of invasive species allelopathy on native plant species varies with biotic and abiotic interactions in the soil (Inderjit & Callaway, 2003). The role of allelopathy in field experiments can be highly context dependent based on the media in which the relationship is tested, as soils may lessen the impact of allelopathy (Harnden, Macdougall & Sikes, 2011). For instance, soil microorganisms can intercept allelochemicals and degrade them into compounds that could be more or less harmful to native plant species (Inderjit, 2005). There may also be allelochemical sorption, transformation, or other interactions with soil nutrients that alter the relative impacts on native species (Inderjit & Weiner, 2001). Indeed, allelochemicals can release metals from chelated compounds, and where metals are limiting, there could be a stronger impact of allelopathy on native species (Barto & Cipollini, 2009). It is important to identify if invasive species are allelopathic to understand their role in structuring the plant community (Thijs, Shann & Weidenhamer, 1994), and using field collected soils from invaded areas can demonstrate an ecologically relevant response.

Here, we explore the context-dependency of allelopathy in an invasive species that is an increasingly problematic species in early successional ecosystems in the eastern United States, *Pyrus calleryana* Decne (Callery pear; Vincent, 2005). *Pyrus calleryana* is generally resistant to pests and pathogens, such as fire blight, and can thrive in degraded soils, creating the potential for widespread success in a variety of environmental conditions (Culley & Hardiman, 2007). This tree has the potential to decrease soil pH due to its acidic litter and may increase soil C:N, indicating that it is altering soil chemistry where it invades (Woods, Attea & McEwan, 2021; Woods, Dietsch & McEwan, 2022a). Further, where *P. calleryana* is established, there is lower cover by native species, and it is possible that this tree could utilize allelopathy as a mechanism to reduce native plant species success (Woods, Dietsch & McEwan, 2022a). However, to our knowledge, there are no documented interactions between *P. calleryana* plant material and native plant germination or success, so its potential allelopathic interactions are unknown.

*Pyrus calleryana* is known for its reliable copious blooming each spring that results in substantial litter from spent flowers (MJ Woods, pers. obs., 2019). There are several examples of flower material harboring allelochemicals and negatively influencing surrounding plants (*e.g.*, Tatari et al., 2010; Singh, Singh & Singh, 2015; Kamble & Pawar, 2020), suggesting that testing for allelopathic effects in plants with substantial floral litter is necessary to fully understand the phenology of allelopathy. Additionally, invasive species commonly have extended leaf phenology where leaves senesce later in the fall than native species (Fridley, 2012), which is also true for *P. calleryana* (Maloney et al., 2022). This phenological pattern could suggest a pattern where leaf material deposited in early winter and may freeze prior to decaying, to thaw in late winter or early spring when the ground thaws. This would lead to potential allelopathic interactions from leaf material well into the spring. Then, in mid to late spring, *P. calleryana* blooms will senesce leading to another potential opportunity for allelopathic interaction.

Our objective was to assess the allelopathic potential of *P. calleryana*, and to gain insight on the context dependency of its potential allelopathy. To test the overall hypothesis that allelopathy is a component of the invasion biology of *P. calleryana* we conducted an experiment testing the effects of *P. calleryana* leaf leachate and flower leachate on the germination of six common prairie species. We assessed the impacts of leaf leachate and flower leachate as well as the potential interaction of leachate with either sterilized sand or soil collected from a *P. calleryana* invaded field site. We predicted that extracts from *P. calleryana* leaf litter and flowers would cause a reduction in seed germination for target species, suggesting that the phenology of this invader could influence its allelopathy. Finally, because soil chemistry can interact with allelochemicals in a way that reduces their influence on germination (*e.g.*, Dalton, Blum & Weed, 1989), we predicted that allelopathic effects would be more apparent in sand than in soil since soils typically have more organic matter that can intercept allelochemicals.

## Materials & Methods

We tested the impacts of *P. calleryana* leaf and flower leachate on the germination of seeds from three grass and three forb species that are commonly used in grassland restorations where seeds often overwinter before spring or late summer germination. All seeds were purchased from Prairie Moon Nursery (Winona, MN, USA). The grass species used were *Sorghastrum nutans* (Indiangrass)*, Elymus canadensis* (Canada wild rye), and *Schizachyrium scoparium* (little bluestem), and the forb species were *Coreopsis lanceolata* (lanced-leaved coreopsis)*, Rudbeckia hirta* (black-eyed Susan), and *Ratibida pinnata* (yellow coneflower). We tested the influence of *P. calleryana* leaf and flower leachate on each species using distilled water as a control. Additionally, we tested the context dependency of this relationship by using both sterile sand and sterile field collected soil as germination media. Thus, we created a full factorial design with three liquid treatments (leaf leachate, flower leachate and DI water) and two soil treatments (sterile field collected soil and sterile sand) with six different species (*n* = 10 plates per species per treatment), accounting for 360 total plates.

### Collection and preparation of materials

We collected soils from the University of Dayton’s Environmental Research Area (UDERA: 39°43′56.3″N, 84°11′27.6″W), a reclaimed and unmanaged field site with a history of sports and recreation that had an established *P. calleryana* invasion in September 2021. These soils were used as an ecologically relevant medium for seed germination. We compared soil media to commercially available sand. The soil was dried and sterilized at 120 °C for 24 h and screened through a two mm sieve prior to use. Sand was sterilized at 120 °C for one hour prior to use. We filled petri plates (100 × 15 mm) with 40 ml of one prepared medium, 180 plates with sand and 180 plates with soil. Soil pH was 7.8 and sand pH was 7.7.

We harvested *P. calleryana* leaves that were at fall color but had not yet senesced in November 2019 and flowers in April 2020 from the UDERA and stored them at −20 °C until creating leachate in September 2021. We made leaf and flower leachate following modified protocols from Dorning & Cipollini (2006) and Bauer et al. (2012). To make the leachate, we incubated whole leaves and whole flowers in DI water for 72 h, with 5 ml of water for each gram of plant material at 4 °C. We vacuum filtered the extract and stored it at 4 °C while plates were prepared. The flower leachate had a pH of 5.5 and the leaf leachate had a pH of 4.5. We added 10 ml of liquid treatments to petri plates, 120 received leaf leachate, 120 received flower leachate and 120 received DI water.

### Germination experiment

We placed ten seeds from one species into ten plates and repeated this ten times for every treatment (liquid × soil). Following species germination requirements, grass species plates were kept at room temperature (∼22 °C) on 12/12 hr day and night cycles directly after seeds were added to each plate. For forb species germination, we cold stratified the plates after seeds were added at 4 °C for thirty days before they were placed in constant room temperature on 12/12 hr day night cycles for 21 days. For each species, we quantified germination every day for 21 days after initiating germination. We refer to seeds that have completed germination as “germinated” throughout the remaining text.

### Statistical methods

All data is publicly available (Woods et al., 2022b). We subset data by species and ran each analysis for each species. We assessed how germination rates change over time by testing the interaction of germination day, germination media and *P. calleryana* treatments on the percent of seeds germinated using a repeated measures ANOVA and including seed plate ID as a random factor. Then, we compared total germination at the end of the experiment by assessing the differences between the interaction of germination media and *P. calleryana* treatments on the percentage of seeds that germinated for each plate after 21 days using ANOVA and Tukey HSD post hoc comparisons. All analyses were performed in R version 4.1.1 (R Core Team, 2021) and data visualizations were created in *ggplot2* (Wickham, 2009).

## Results

*Pyrus calleryana* leaf and flower leachate reduced the germination of five out of six study species when incubated in sand. For three of our study species, leachate and soil type interacted to determine seed germination, where germination was consistently higher in the combination of sand and water. For two species, both leachate and soil exhibited strong main effects with increased germination in water, but the responses were species-specific, and no interactions were detected between leachate and media. For one species, there was no effect of leachate on germination, and germination occurred more often in field collected soil than in sand.

### Sorghastrum nutans

**Figure 1:**
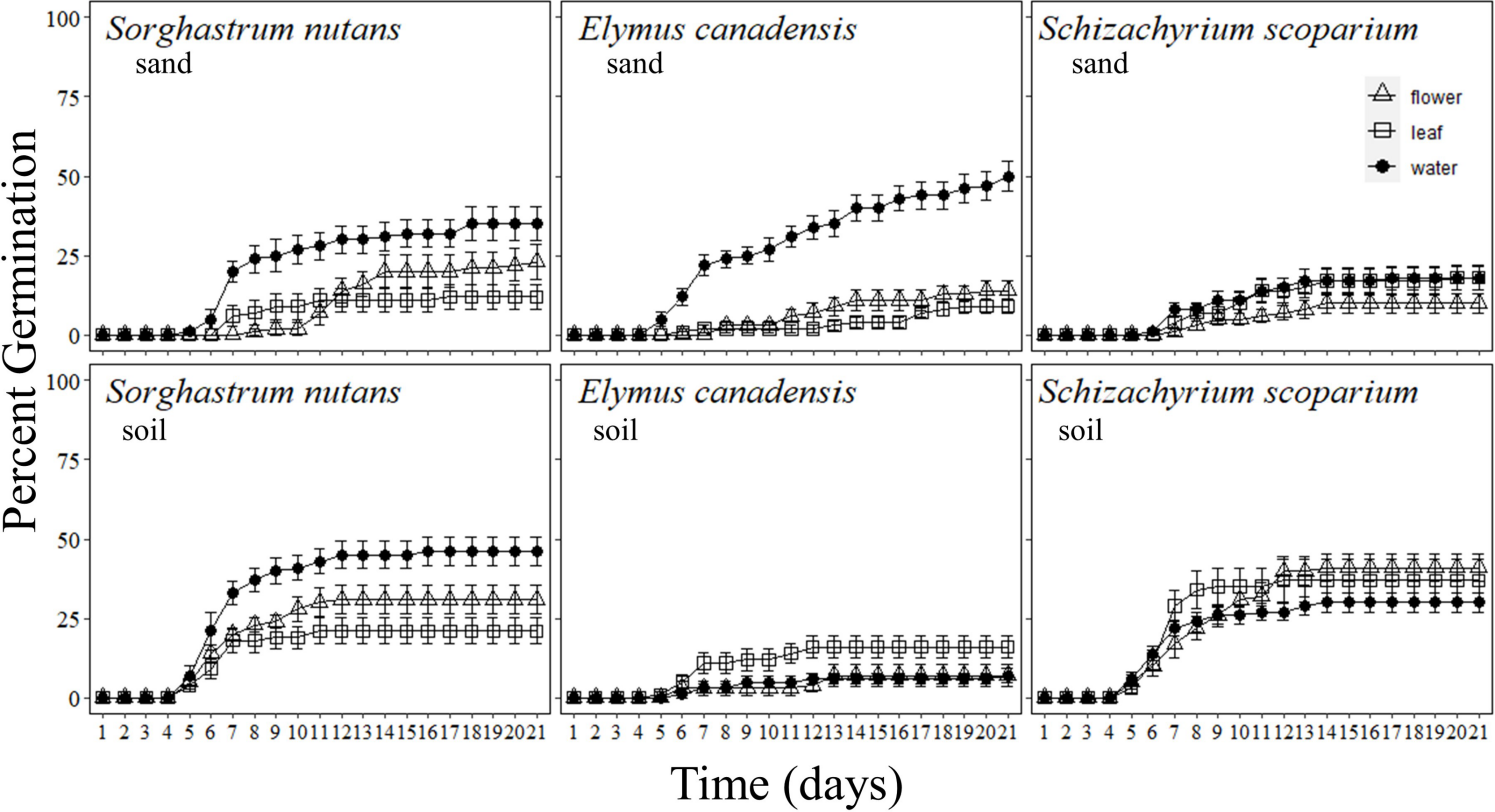
Percentage of grass seeds that germinated with *Pyrus calleryana* leachate in either sand or soil. Percentage of seeds that germinated from three commonly planted prairie grass species that were germinated with either sand (top row) or soil (bottom row) and treated with water (black circle), *Pyrus calleryana* leaf leachate (white square) or *P. calleryana* flower leachate (white triangle). Each point represents the average percentage germinated per day and bars represent standard error.

*Sorghastrum nutans* germination was reduced by exposure to both flower and leaf leachate from *P. calleryana* (*F*_40,1080_ = 3.065, *P* < 0.001; Fig. 1). By seven days, germination of seeds incubated in soil and water was significantly higher than those incubated in leachate from *P. calleryana* and this continued until the end of the experiment. Additionally, after one week, seeds incubated in sand and leaf leachate or sand and flower leachate had the lowest percentage of germinated seeds. By the end of the experiment, *S. nutans* germinated more often in soil than sand (*F*_1,58_ = 4.342, *P* = 0.0416; SI Fig. 1) and in water than either flower or leaf leachate (*F*_2,57_ = 12.72, *P* < 0.001; SI Fig. 1). There was not a statistically significant interaction between the incubation media and liquid treatment for *S. nutans* (*P* > 0.05).

### Elymus canadensis

*Pyrus calleryana* extracts reduced the germination of *E. canadensis* in sand such that it germinated faster with sand and water than any other treatment (*F*_40,1080_ = 18.582, *P* < 0.001; Fig. 1). By day seven, an average of 22% of seeds incubated with sand and water germinated with 11% of seeds incubated in leaf leachate and soil germinated and 2–3% of all other treatments germinated. By the end of the experiment, an average of 50% of seeds germinated with sand and water, but all other combinations of treatments had indistinguishable germination rates with an average of 10% germination including every soil treatment (*F*_2,54_ = 31.03, *P* < 0.001; SI Fig. 2).

**Figure 2:**
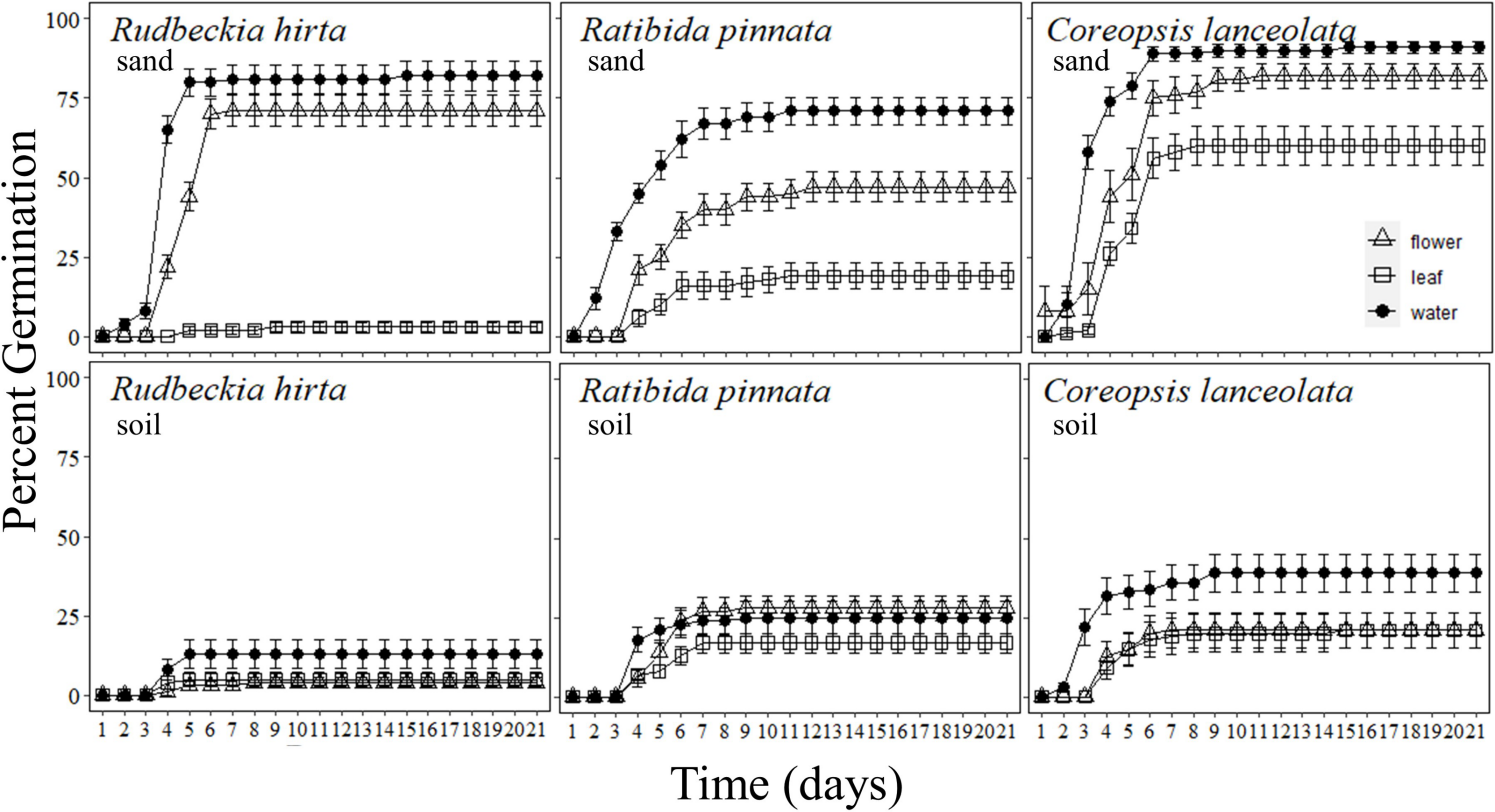
Percentage of forb seeds that germinated with *Pyrus calleryana* leachate in either sand or soil. Percentage of seeds that germinated from three commonly planted prairie forb species that were germinated with either sand (top row) or soil (bottom row) and treated with water (black circle), *Pyrus calleryana* leaf leachate (white square) or *P. calleryana* flower leachate (white triangle). Each point represents the average percentage germinated per day and bars represent standard error.

### Schizachyrium scoparium

The germination rate of *S. scoparium* was not influenced by *P. calleryana* extracts in either sand or soil. Overall, germination occurred earlier when seeds were incubated in soil than in sand (*F*_40,1080_ = 3.065, *P* < 0.001; Fig. 1). By the sixth day of germination, there was a statistically significant difference between sand and soil with an average of 4.6% germinated in soil and 0% germinated in sand. By 21 days, an average of 36% of *S. scoparium* seeds germinated in soil and 15.3% germinated in sand with no statistically significant differences between liquid treatments (*F*_1,58_ = 34.2, *P* < 0.001; SI Figure 3).

### Rudbeckia hirta

*Rudbeckia hirta* germinated markedly faster in sand with water or *P. calleryana* flower leachate than any other combination of treatments (*F*_40,1080_ = 46.48, *P* < 0.001; Fig. 2). By day 5 of germination, an average of 80% of seeds incubated in sand with water had germinated, 44% of seeds with sand and flower leachate had germinated, 13% of seeds with soil and water had germinated, with less than 5% germination of each other treatment. By day 7, the final germination rates were met with an average of 82% germinated in sand water, 71% in sand flower leachate and 13% in soil and water, and still less than 5% germination in either media with leaf leachate or soil and flower leachate. Thus, there was an interaction between germination media and *P. calleryana* treatment such that when *R. hirta* seeds were incubated with sand and flower leachate or water, they had increased germination occurrence than all other treatments (*F*_2,54_ = 65.28, *P* < 0.001; SI Figure 3).

### Ratibida pinnata

*Ratibida pinnata* germinated at the highest rate when incubated with sand and water, followed by sand and *P. calleryana* flower leachate, with an intermediate germination rate for soil and water and soil and flower leachate, while leaf leachate caused the slowest germination regardless of germination media (*F*_40,1080_ = 5.375, *P* < 0.001; Fig. 2). The rate of germination was highest for seeds incubated with sand and water, with an average of 12% of seeds germinated and 0 seeds germinated for other treatments. By 21 days of germination, an average of 71% of seeds germinated in water and sand, 47% of seeds germinated in flower leachate and sand, an average of 26% of seeds germinated in soil with flower leachate and soil with water, and finally 17% germinated in soil with leaf leachate and 19% germinated in sand leaf leachate. This pattern was observed by day 7 of germination. Finally, more *R. pinnata* seeds germinated in sand and water than any other treatment combination, followed by sand and flower leachate, but seeds incubated in sand with leaf leachate and all seeds in soil media had lower but indistinguishable levels of overall germination (*F*_2,54_ = 13.83, *P* < 0.001; SI Fig. 1).

### Coreopsis lanceolata

*Pyrus calleryana* leaf and flower leachate reduced germination rates of *C. lanceolata*, with the highest germination present in the sand and water treatment (*F*_40,1080_ = 2.150, *P* < 0.001; Fig. 2). By day 3 of germination, seeds with sand and water had germinated an average of 58%, while all other treatments are statistically indistinguishable with an average of 7.8%. After 7 days, the pattern of germination remained constant until the end of the experiment where an average of 91% of seeds incubated with sand and water germinated, 82% of seeds with sand and flower leachate germinated, 60% of sand and leaf leachate germinated, 39% of soil and water germinated and 21% of soil with flower leachate or leaf leachate germinated. Thus, by the end of the experiment, all seeds in sand germinated more than seeds in soil and those that were incubated with water germinated more than flower or leaf leachate (*F*_1,58_ = 105.5, *P* < 0.001; SI Fig. 1).

## Discussion

Our results indicate that allelopathic suppression of native plants may be one important aspect of *Pyrus calleryana* invasion ecology. *Pyrus calleryana* reduced the germination of five out of six native grassland species tested in this laboratory study, with stronger effects of leaf leachates than flower leachates. This could indicate that the constituent secondary metabolites found in *P. calleryana* differ by the plant material assessed, or that the harmful metabolites are concentrated within the leaves. This experiment demonstrated the importance of testing allelopathic potential of different plant material to determine the phenological impact of plant invasion on native species. Further, allelopathic potential of *P. calleryana* depended on the substrate that seeds were incubated in, which suggests that its allelopathy is context dependent, and in order to understand the impacts of *P. calleryana* on native plant species, the soils must be considered.

One unique finding in this study is that *P. calleryana* flower extracts inhibited germination for some species. This finding may suggest the potential for an extended phenology of allelopathy inhibition due to *P. calleryana* invasion. Leaf deposition of this species in autumn is later than native trees (Maloney et al., 2022) and could potentially inhibit seed germination in early spring if they freeze over winter. A second pulse of flower litter in late spring could extend the window of germination inhibition and could be particularly influential in Eastern US grasslands where seed germination of many species occurs in mid spring (Blake, 1935). There are several instances of other plant species exhibiting allelopathic impacts *via* flower litter, such as *Cassia fistula* which decreases the germination of *Alternanthera tenella* (Kamble & Pawar, 2020) and *Nicotiana plumbaginifolia* inhibiting germination of *Helianthus annuus* (Singh, Singh & Singh, 2015). Additionally, *L. maackii* flower litter reduced the survival of the macroinvertebrate *Hyalella azteca* in stream assays (Custer et al., 2017), demonstrating that deposition of invasive plant flower litter is ecologically important in some contexts. Overall, our work demonstrates that *P. calleryana* leaf litter has allelopathic potential and that flower litter deposition in late spring may result in a second dose of allelochemicals into the soil system.

Germination rates responded to incubation media, either sterilized field collected soil or sterilized sand, and indicated that *P. calleryana*’s allelopathic potential is likely mediated by environmental conditions. For instance, most species germinated more often in sand than soil, when water was used as a liquid treatment. This was surprising because soils typically have more organic matter than sand, and organic matter can lead to sorption of allelochemicals and can alleviate the negative impacts of allelopathy (Dalton, Blum & Weed, 1989; Kobayashi, Koyama & Shim, 2004; Tharayil et al., 2009). Further, two grass species, *S. nutans* and *S. scoparium* had higher germination rates in soil than sand, and *S. scoparium* germination was not altered by *P. calleryana* leachate. A pattern of overabundant grasses and reduced forb diversity in restored grasslands has been documented throughout the US (Grman et al., 2021) and since *P. calleryana* leachate reduces forb germination more severely than grass germination, this invasion could exacerbate this pattern, leading to reduced diversity during restoration. These results also suggest that substrate may regulate the role of allelopathy during plant invasions and understanding the interactions of allelochemicals with soil is essential for interpreting the potential allelopathic impacts of invasive species. Notably, our study only assessed sterilized soils, removing biological interactions of soil microorganisms and allelopathic chemicals. It is possible that allelopathic potential could be inhibited or exacerbated by microorganism metabolism (Inderjit, 2005).

This study documents one possible mechanism driving the competitive success of *P. calleryana*, an aggressive invader throughout the Eastern US. *Pyrus calleryana* reduced germination for five out of six tested species in this study, suggesting that it is likely allelopathic. The flowers of this tree may also contain allelochemicals that inhibit native species germination, indicating that the relative impacts of allelopathy could be driven by this plant’s phenology. One implication of these results is that control activities that seek to remove *P. calleryana* should take place prior to flowering in the spring to avoid deposition of potentially toxic flower litter. Selective effects of *P. calleryana* allelopathy may act as a filter by reducing the germination and establishment of forb species but could have little to no effect on overabundant grass species that may become dominant within grasslands. *Pyrus calleryana* is an effective invader of open habitats in eastern North America and allelopathy effects may partially explain why invasion is associated with reduced native plant cover (Woods, Dietsch & McEwan, 2022a).

## Conclusions

In this study, we identified that the prolific invader *P. calleryana* has allelopathic effects on native species, where it reduced the germination of five out of six tested grassland species. Further, we found that the strength of this allelopathic relationships differed by the plant material assessed, suggesting that the allelopathic chemicals present in the leaf and flower material of *P. calleryana* may differ. The potential for the flowers to be allelopathic also may extend the window in which allelochemicals can leach from *P. calleryana* plant material into the soil, extending the window of opportunity of reduced plant germination from leaf senescence in the late fall to flower senescence in the late spring. Finally, the germination rates of the grassland species were higher in sand than soil, suggesting that the soils in which germination is occurring can act as a mediator of allelopathy and there may be context dependency of this relationship that depends on soil chemistry. It may be important to note that *P. calleryana* exhibits a high level of genetic diversity throughout its invaded range (Sapkota et al., 2022), suggesting that allelopathic effects may also depend on their genetic background. Thus, to better understand the allelopathic potential of *P. calleryana,* the allelochemicals found in its plant litter should be identified and the interactions between these chemicals and the soil habitat should be considered, such as the potential for sorption by organic matter or breakdown by microorganisms.

##  Supplemental Information

10.7717/peerj.15189/supp-1 Supplemental Information 1 Supplemental Figures

## Additional Information and Declarations

### Data Availability

The following information was supplied regarding data availability: The data is available at the University of Dayton: Woods, Michaela J.; Bauer, Jonathan T.; Schaeffer, Dena; and McEwan, Ryan W.. Callery Pear Allelopathy Study. Five Rivers MetroParks Collaboration Data Archive [dataset]. Retrieved from https://ecommons.udayton.edu/mcewanlab_3_data/10.
