# Supplementary material for: Pyrus calleryana extracts reduce germination of native grassland species, suggesting the potential for allelopathic effects during ecological invasion
Source: PeerJ. 2023 Apr 25;11:e15189. doi: 10.7717/peerj.15189 (PMC10143590; doi:10.7717/peerj.15189)
Supplement: Supplemental Information 1 [file peerj-11-15189-s001.docx]

**Supplemental Figures**


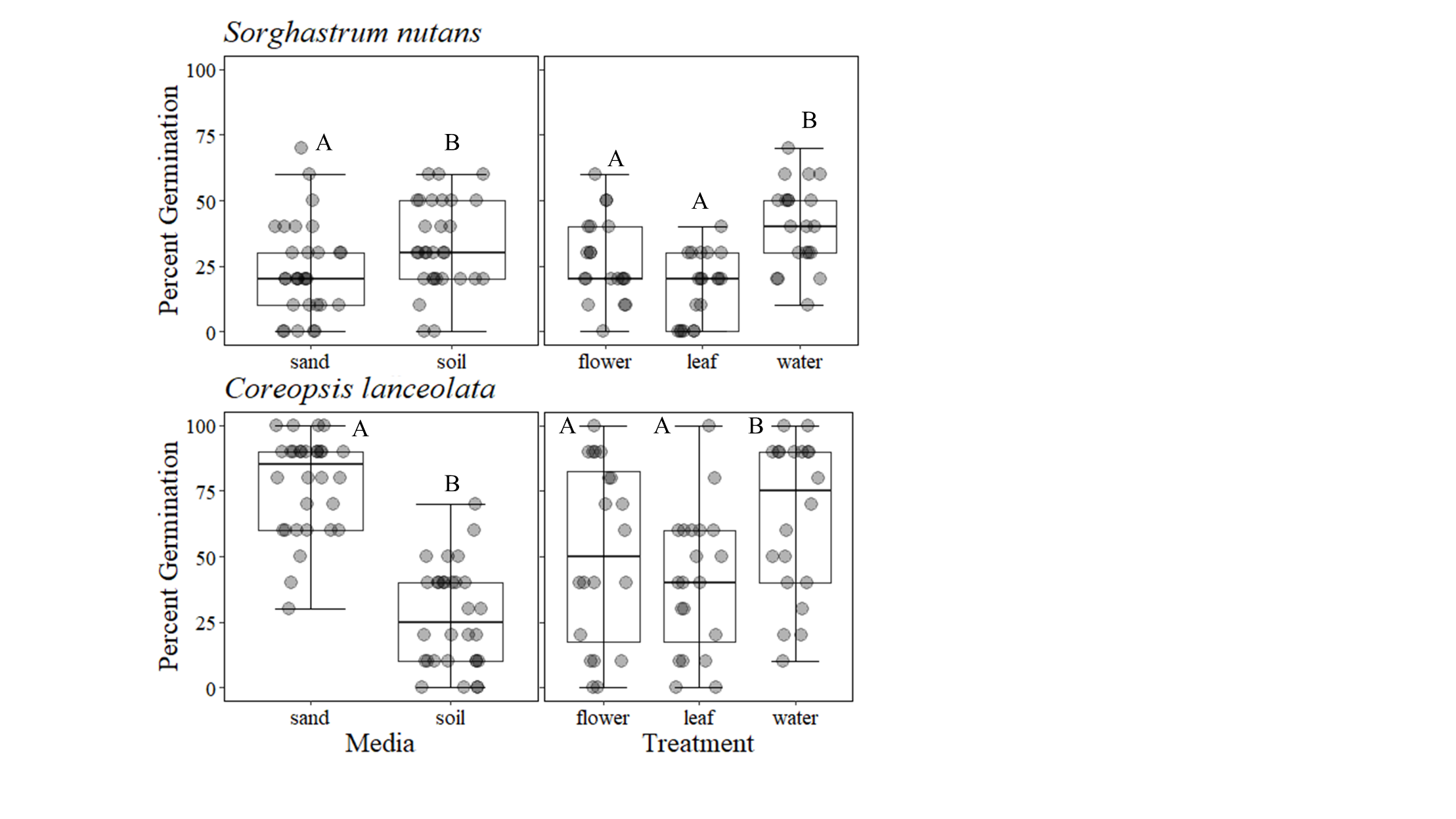

**Figure 1.** The effect of germination media (sand or soil) and the effect of liquid treatment (*Pyrus calleryana* leaf flower leachate, leaf leachate or water) on the percent germination of Indian grass and lance leaf coreopsis. Each point represents one germination plate that has ten seeds after 21 days on incubation.


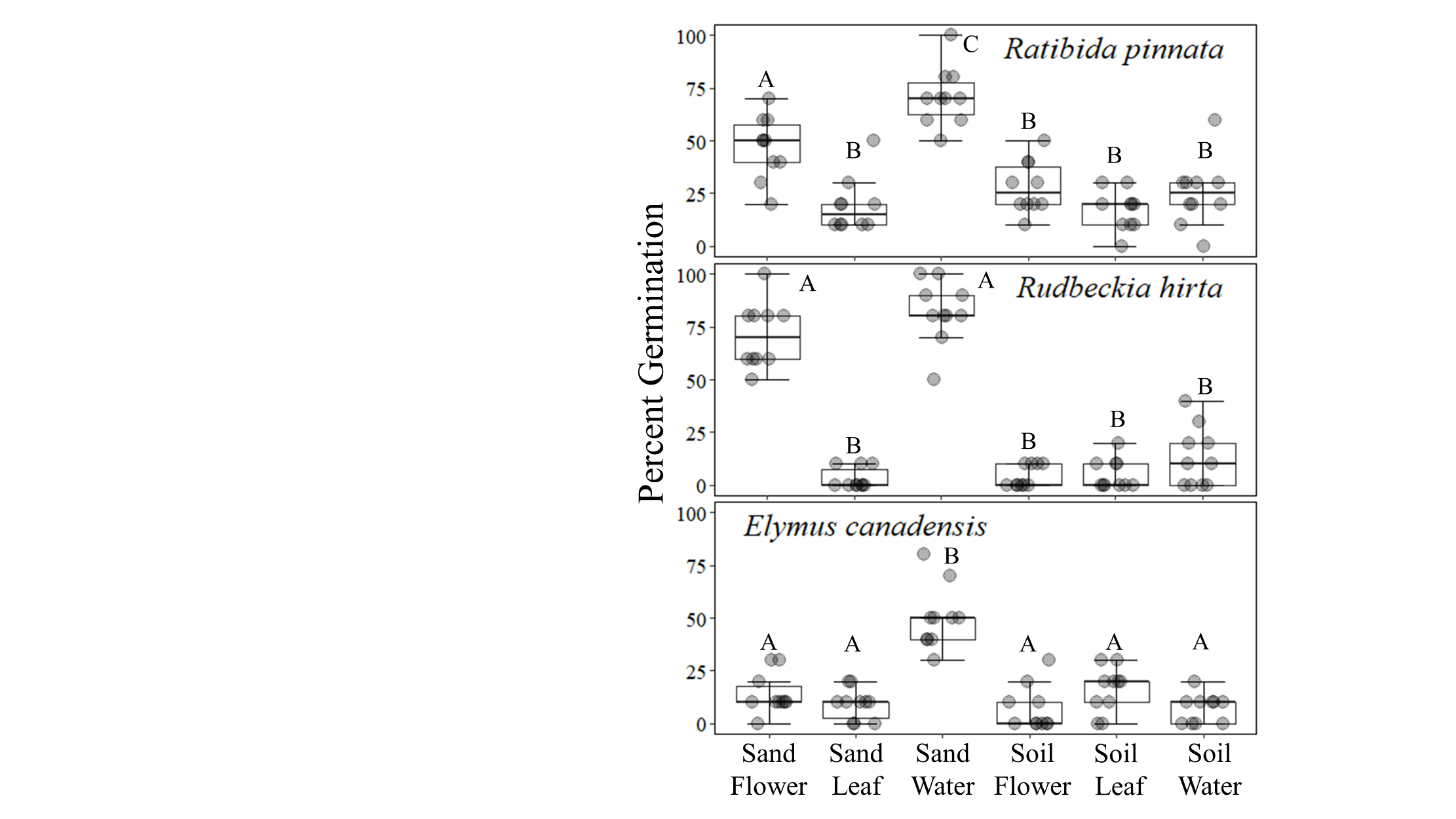


**Figure 2.** The interactive effects of germination media (sand or soil) and liquid treatment (*Pyrus calleryana* flower leachate, leaf leachate or water) on the percent germination of Canada wild rye, black-eyed Susan and yellow coneflower after 21 days of incubation. Each point represents one germination plate containing ten seeds.


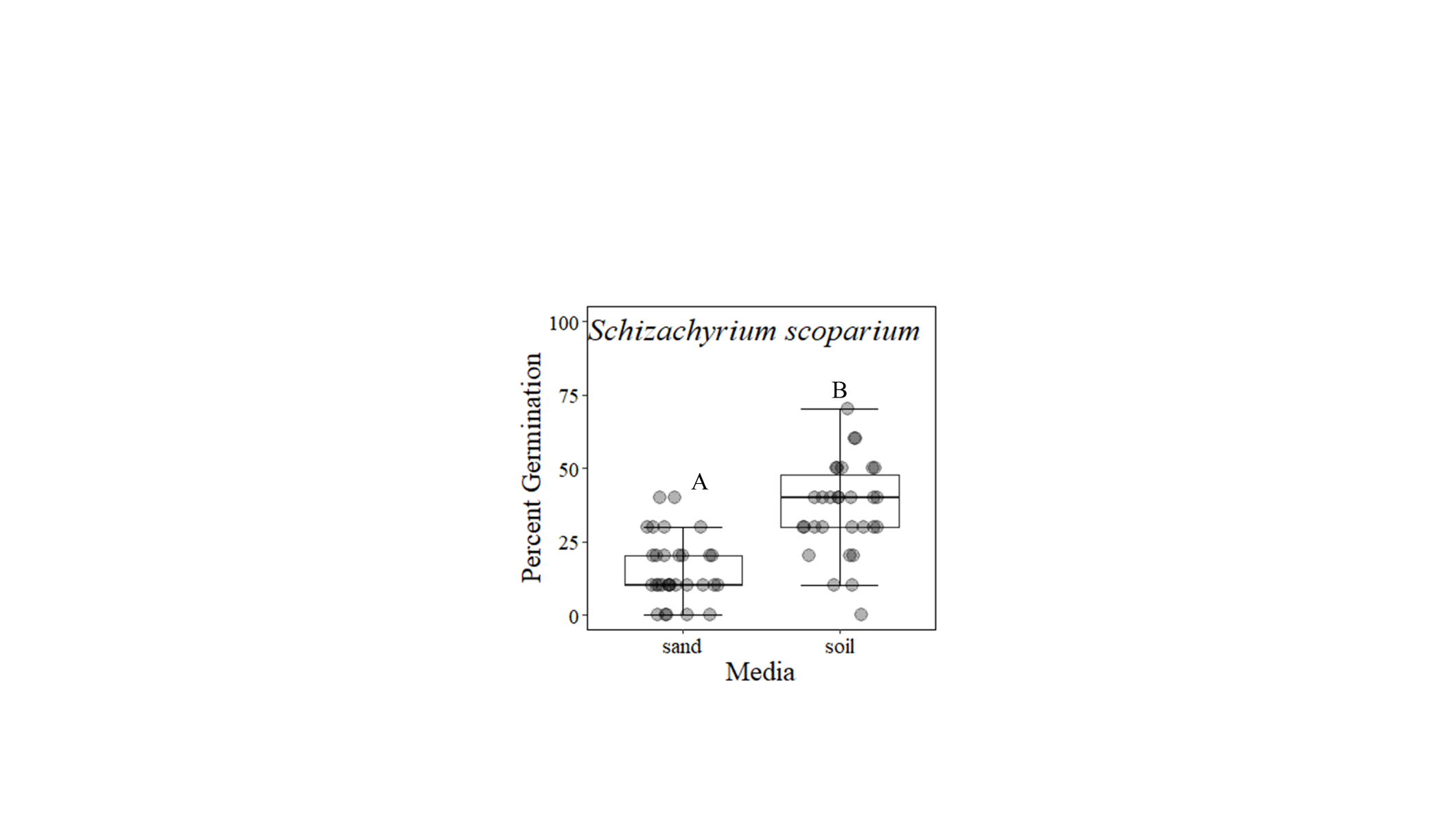


**Figure 3.** The effect of germination media (sand or soil) on the overall percentage of little bluestem seeds to germinate despite being incubated with *Pyrus calleryana* leaf leachate, flower leachate or water. Each point represents one plate of ten germinating seeds after 21 days of germination.
